# Draft genome sequences of 12 fungi occurring in cyanobacteria-dominated biotic soil crusts in the Sonoran Desert

**DOI:** 10.1128/mra.00637-26

**Published:** 2026-08-13

**Authors:** Pablo Sekul Arán, O. Joshua Oyekanmi, Joseph Spraker, A. Elizabeth Arnold

**Affiliations:** 1 Department of Ecology and Evolutionary Biology, The University of Arizona 8041, Tucson, Arizona, USA; 2 Ecosystem Genomics Graduate Interdisciplinary Program, The University of Arizona 8041, Tucson, Arizona, USA; 3 School of Plant Sciences, The University of Arizona 8041, Tucson, Arizona, USA; 4 Hexagon Bio 542226 , Menlo Park, California, USA; 5 BIO5 Institute, The University of Arizona 8041, Tucson, Arizona, USA; University of California Riverside, Riverside, California, USA

**Keywords:** arid lands, Ascomycota, biological soil crust, desert, fungi

## Abstract

We present draft genome sequences for 12 fungal isolates, including two isolates each of *Trichoderma*, *Exophiala*, and *Cladosporium*, and one isolate each of *Fusarium*, *Cladorrhinum*, *Epicoccum*, Chaetomiaceae, Didymellaceae, and Pleosporales, from cyanobacteria-dominated biological soil crusts in the Sonoran Desert (Arizona, USA), sequenced on a NovaSeq 6000 at ≥40× coverage.

## ANNOUNCEMENT

Biological soil crusts (BSCs) are communities of cyanobacteria, algae, fungi, lichens, liverworts, mosses, and other microbes that form cohesive surface layers in arid and semiarid ecosystems. They stabilize soil, promote water retention, facilitate carbon and nitrogen cycling, and support the establishment and growth of vascular plants (1–3). Cyanobacteria-dominated BSCs in the Sonoran Desert harbor diverse fungi for which genomic resources remain limited (4). Here, we report the draft genome sequences of 12 fungal isolates recovered from cyanobacteria-dominated BSCs collected near Tucson, AZ, USA, in an area with upland Sonoran Desert vegetation (32.230662° N, 111.037208° W).

To isolate the fungi, we macerated small fragments of BSC (0.5 cm^2^) in 750 μL of sterile water. Aliquots of the resulting suspension (each 20 μL) were spread onto 2% malt extract agar (MEA). Cultures were incubated at room temperature for up to 3 weeks, with morphologically distinct isolates transferred to axenic culture on 2% MEA.

Isolates were characterized by colony morphology and sequencing of the internal transcribed spacers, the 5.8S gene, and partial nuclear large subunit rDNA (5). They were identified as species of *Trichoderma*, *Fusarium*, *Cladorrhinum*, *Cladosporium*, *Exophiala*, *Epicoccum*, and members of Chaetomiaceae, Didymellaceae, and Pleosporales (Table 1). Some isolates were maintained on antibiotic-amended MEA (kanamycin, chloramphenicol, ampicillin, and tetracycline; see reference 5). Voucher cultures are maintained at the University of Arizona’s Robert L. Gilbertson Mycological Herbarium (Table 1).

**TABLE 1:** Summary of genome assembly statistics for fungi isolated from cyanobacteria-dominated biocrusts in the Sonoran Desert

Fungal isolate	Accession no.	NCBI assembly	Genome size (Mb)	GC content (%)	No. of genes	Coverage	Contigs	N50 (bp)	Total reads (bp)	Genome completeness (%)
*Trichoderma* sp.	444A_sa34508	JBYJVW000000000	39.9	48.0	11,235	124.9×	559	256,671	16,739,005	99.6
*Fusarium* sp.	UA_444B_sa91877	JBYJVX000000000	39.6	48.0	12,175	43.1×	246	915,415	11,455,829	99.5
*Cladorrhinum* sp.	445A_sa34509	JBYJVY000000000	35.9	50.8	10,573	153.3×	941	64,232	18,815,361	99.6
*Trichoderma* sp.	UA_446A_sa91860	JBYJVZ000000000	38.2	49.2	11,075	44.0×	672	134,157	11,476,384	99.8
*Cladosporium* sp.	UA_447A_sa155827	JBYJWA000000000	33.5	54.1	10,445	168.4×	132	444,406	38,584,594	99.8
*Exophiala* sp.	448A_sa27466	JBYJWB000000000	36.8	50.6	16,128	83.3×	462	291,622	11,730,147	99.5
*Exophiala* sp.	449A_sa34510	JBYJWC000000000	32.5	50.3	12,155	159.4×	234	223,311	17,297,610	99.7
*Cladosporium* sp.	UA_450A_sa91895	JBYJWD000000000	54.0	50.6	16,418	194.1×	1592	447,552	73,635,417	99.7
*Epicoccum* sp.	537A_sa34541	JBYJWE000000000	33.1	52.5	11,294	159.4×	355	229,003	17,615,266	99.1
Chaetomiaceae sp.	UA_537B_sa91975	JBYJWF000000000	28.9	49.1	10,408	45.3×	346	274,997	8,912,106	99.9
Didymellaceae sp.	UA_537F_sa91762	JBYJWG000000000	33.1	50.7	10,712	54.7×	484	453,504	12,195,236	99.9
Pleosporales sp.	UA_538A_sa91976	JBYJWH000000000	31.1	49.6	10,185	75.7×	500	137,612	16,440,456	99.7

For DNA extraction, strains were grown on 2% MEA amended with 0.5% yeast extract for 14 days at 25°C. Mycelia were harvested, frozen at −80°C, lyophilized, and homogenized by bead beating (6). Genomic DNA was extracted with the ZymoBIOMICS Soil Kit (Zymo Research, Irvine, CA, USA). For library preparation, 25 ng of total genomic DNA was processed with the KAPA HyperPlus Kit (Roche, Switzerland), amplified for eight cycles, pooled, size-selected (350–900 bp) via the Zymo Select-a-Size Clean & Concentrator Kit and gel purification.

Whole genomes were sequenced using the NovaSeq 6000 platform (Illumina, San Diego, CA, USA), generating 151 bp paired-end reads at ≥40× coverage. Adapter trimming and low-quality reads were filtered with fastp (v0.21.0) (7). Genomes were assembled with SPAdes (v3.15.4) with default parameters (8). For taxonomic identification, assembled genomes were compared against the NCBI fungal genome database via BLASTn (v2.17.0) with default parameters, and the top-scoring alignments were used for species assignments. Annotations were completed with BRAKER3 (v3.0.8) (9, 10) with only protein models from the closest relatives available in the Joint Genome Institute MycoCosm database. Assembly completeness was assessed using BUSCO (v6.1.0) with the fungi_odb10 lineage data set (11). We predicted secondary metabolite biosynthetic gene clusters (BGCs) using antiSMASH (v8.0.4) (12).

Genome assembly statistics for the 12 strains are summarized in Table 1. Based on antiSMASH ClusterBlast comparisons against the MIBiG database, predicted BGCs were similar to metachelin C/A/B/dimerumic acid siderophore BGCs and several previously characterized BGCs associated with metabolites implicated in UV protection, oxidative stress tolerance, and adaptation to nutrient-limited environments (13, 14). BRAKER3 annotation (.gff) and antiSMASH output files are available at https://doi.org/10.5281/zenodo.19137085.

## Data Availability

Genome sequence data are available at GenBank under BioProject accession number PRJNA1425102 and BioSample accession numbers SAMN55406242–SAMN55406253. Individual genome assemblies are available under accession numbers JBYJVW000000000–JBYJWH000000000 (see Table 1). Raw sequencing reads are available in the Sequence Read Archive under accession number PRJNA1425102. Additional data on BGCs are available at https://doi.org/10.5281/zenodo.19137085.
